# Claudin-10 overexpression suppresses human clear cell renal cell carcinoma growth and metastasis by regulating ATP5O and causing mitochondrial dysfunction

**DOI:** 10.7150/ijbs.70105

**Published:** 2022-03-06

**Authors:** Wuping Yang, Kenan Zhang, Zedan Zhang, Jingcheng Zhou, Lei Li, Yawei Xu, Jianhui Qiu, Lin Cai, Yanqing Gong, Kan Gong

**Affiliations:** 1 Department of Urology, Peking University First Hospital, Beijing 100034, P.R. China.; 2 Hereditary Kidney Cancer Research Center, Peking University First Hospital, Beijing 100034, P.R. China.; 3 Institute of Urology, Peking University, Beijing 100034, P.R. China.

**Keywords:** claudin-10, ATP5O, acetylation, mitochondria dysfunction, NDUFS2

## Abstract

Our previous study has proved that down-regulation of CLDN10 (Claudin-10) in ccRCC (clear cell renal cell carcinoma) was closely related to tumor metastasis and predicted an unfavorable prognosis by analyzing TCGA-KIRC data. However, the effects of CLDN10 on the progression of ccRCC and its mechanisms of action remain elusive. During the study, a large number of clinical samples were utilized to verify the reduced expression of CLDN10 in ccRCC and its association with tumor metastasis and poor prognosis, and our results confirmed that lower CLDN10 expression was an independent predictor of shorter OS (HR: 4.0860, 95%CI: 2.4737-6.7490, P<0.0001) and DFS (HR: 4.3680, 95%CI: 2.2800-8.3700, P<0.0001) in metastatic ccRCC patients. CLDN10 overexpression accelerated cell apoptosis and restrained cell proliferation, migration and invasion *in vitro*. Besides, CLDN10 overexpression suppressed ccRCC growth and lung metastasis and promoted apoptosis in orthotopic models. Mechanistically, we found that CLDN10 overexpression up-regulated the acetylation and expression levels of ATP5O (ATP synthase subunit O, mitochondrial), leading to the dysfunction of mitochondrial, thereby suppressing the growth and metastasis of ccRCC through increasing the levels of NDUFS2, ROS, Cleaved-Caspase 3, E-cadherin and SDHB and decreasing the levels of N-cadherin and mitochondrial membrane potential. Moreover, knockdown of ATP5O expression based on the overexpression of CLDN10 could reverse the increase in NDUFS2, ROS, Cleaved-Caspase 3, E-cadherin and SDHB levels, the decrease in N-cadherin and mitochondrial membrane potential levels and the inhibition of ccRCC phenotypes caused by CLDN10 overexpression. Taken together, these findings for the first time illuminate the mechanism by which CLDN10 overexpression suppresses the growth and metastasis of ccRCC.

## Introduction

Kidney cancer has caused approximately 430,000 new cases and nearly 180,000 deaths in 185 countries in 2020 1. RCC (Renal cell carcinoma) accounts for the majority of kidney cancer, and ccRCC (clear cell RCC) is the most common subtype of RCC, accounting for 75% to 80% of RCC 2. Moreover, its incidence has increased significantly in recent years 3. Although the 5-year survival rate of patients with early RCC has reached more than 90% due to advances in surgical methods and the emergence of more therapeutic drugs, the survival rate of patients with advanced metastatic tumor is as low as below 10% 4, 5. One of the important reasons for this is that patients with mRCC (metastatic RCC) have already lost the opportunity for surgery, and drug therapy is one of the most important treatment options for most patients at this stage. Recent studies have also proposed some new mechanisms for tumor metastasis, which may contribute to the discovery of new therapeutic targets. For example, NONO (non-POU domain-containing octamer-binding protein) inhibited lymphatic metastasis of bladder cancer through alternative splicing of SETMAR 6; amplification of the inherent inflammation of cancer cells triggered neutrophil-dependent lung metastases during the progression of ccRCC 7. Nevertheless, the current drug treatments for mRCC patients are not effective enough, and drug resistance is easy to develop during the treatment process 8-10. Hence, it is of great importance to continuously explore new mechanisms of renal cancer metastasis and to find novel targets to inhibit the progression of renal cancer.

Claudins are the pivotal transmembrane proteins in TJs (tight junctions), and their expression are often reduced and play important roles in ccRCC, including CLDN8 (claudin-8) 11, CLDN7 (claudin-7) 12, and CLDN2 (claudin-2) 13. Previous studies reported that CLDN10 (claudin-10), as a member of the claudins family, played a vital role in different tumors, including hepatocellular carcinoma 14, papillary thyroid cancer 15, and gastric cancer 16. However, there are few studies on the roles of CLDN10 in kidney cancer. Although our previous study has indicated that CLDN10 was down-regulated in ccRCC and decreased expression of CLDN10 was closely associated with ccRCC metastasis and poor prognosis, most of these findings were gained by analyzing TCGA-KIRC data 17. Thus, more clinical samples are needed to clarify the specific clinical roles of CLDN10 in ccRCC, and more basic experiments are required to determine the specific mechanism of CLDN10 inhibiting ccRCC metastasis to provide important theoretical support for the exploitation of a novel kidney cancer therapeutic target.

ATP5O protein (ATP synthase subunit O, mitochondrial) is a component of ATP synthase (F1F0 ATP synthase or Complex V) found in the mitochondrial matrix. Previous studies have reported that the expression of ATP5O played an important role in the diagnosis and prognosis of gastric cancer 18, and analysis results of NextBio database also showed that ATP5O gene expression was down-regulated in ccRCC 19. SIRT3 (NAD-dependent protein deacetylase sirtuin-3, mitochondrial) is considered to be a deacetylase of ATP5O 20, 21. SIRT3 can play the role of both oncogene and tumor suppressor gene in different tumors. For instance, SIRT3 expression was an independent predictor of esophageal cancer treatment, and the higher the expression, the worse the prognosis 22; SIRT3 promoted survival and protected cells from damage by regulating apoptotic factors in fibrosarcoma, cervical cancer, bladder cancer and oral squamous cell carcinoma 23-25, overexpression of SIRT3 restrained the growth of kidney tumor cells and enhanced mitochondrial biogenesis 26.

In this study, we used immunohistochemistry and western blot to clarify the expression pattern of CLDN10 and its prognostic roles in clinical ccRCC and mccRCC samples. TUNEL, EdU, flow cytometry, cell migration and invasion experiments were used to determine the biological function of CLDN10 in ccRCC. In terms of mechanism, TMT (Tandem mass tags), immunofluorescence and Co-IP experiments were utilized to explore the molecular mechanism of CLDN10 suppressing the growth and metastasis of ccRCC. Besides, we further verified the inhibitory effect of CLDN10 on the growth and metastasis of ccRCC through orthotopic models. In the end, we concluded that CLDN10 overexpression could inhibit the growth and metastasis of ccRCC by regulating ATP5O acetylation and expression levels to alter its downstream proteins and pathways.

## Materials and Methods

### Ethics statement

This study was approved by the Ministry of Science and Technology of the People's Republic of China (2021SLCJ2189). Informed consent signed by each patient has been obtained in this study.

### TCGA-KIRC data acquisition

RNA-sequencing data of ATP5O and NDUFS2, and the clinicopathological data of ccRCC patients were gained from TCGA-KIRC (The Cancer Genome Atlas-Kidney Renal Clear Cell Carcinoma). The correlation between ATP5O expression and OS (Overall Survival), the correlation between NDUFS2 expression and DFS (Disease Free Survival), the correlation between CLDN10 and ATP5O and NDUFS2 expression, and the correlation between ATP5O and NDUFS2 expression were realized through the GEPIA online website.

### Clinical samples collection

Samples from 54 patients diagnosed with ccRCC and 122 patients diagnosed with mccRCC were included in the study. All samples were provided by the Department of Urology, Peking University First Hospital. Clinicopathological information and prognostic data of the 54 ccRCC and 122 mccRCC cases were also obtained.

### Cell culture

The normal human renal tubular epithelial cell line HK2, human embryonic kidney cell line HEK293 and five ccRCC cell lines 786-O, 769-P, OSRC2, A498 and Caki-1 were used in the present study. All cell lines were obtained from the American Type Culture Collection (Rockville, MD, USA). CLDN10 overexpression (with 3xflag), ATP5O overexpression (with 3xflag) and ATP5O knockdown plasmids were constructed by the SyngenTech Company (SyngenTech Co. Ltd., Beijing, China). Cells were transfected with the corresponding vector using Lipofectamine 3000 Transfection Reagent (Invitrogen, USA) according to the manufacturer's instructions. The stable cell lines were established by lentivirus infection accordingly.

### Western blot

Total protein was obtained using RIPA lysis buffer. Cell membrane, cytoplasm and mitochondrial proteins were obtained using the cell membrane protein and cytoplasmic protein extraction kit (Beyotime) and the cell mitochondrial isolation kit (Beyotime). BCA protein assay Kit (APPLYGEN) was used to quantitate the protein levels. The primary antibody information used is as follows: anti-CLDN10 (1:1000; Abcam, ab52234), anti-Flag (1:1000; CST, 14793S), anti-ATP5O (1:2000; Abcam, ab110276), anti-Acetyl-ATP5O (1:200; Abcam, ab214339), anti-SIRT3 (1:1000; Abcam, ab217319), anti-NDUFS2 (1:5000; Abcam, ab192022), anti-Cleaved-Caspase 3 (1:1000; Affinity, AF7022), anti-E-cadherin (1:10000; Abcam, ab40772), anti-N-cadherin (1:5000, Abcam, ab76011), anti-SDHB (1:5000, Proteintech, China), anti-GAPDH (1:8000, Proteintech, China), anti-ATP1A1 (1:8000, Proteintech, China) and anti-COX Ⅳ (1:8000, Proteintech, China).

### Immunohistochemistry

The paraffin sections of ccRCC and mccRCC tissues were used to perform immunohistochemical staining to measure the protein levels of CLDN10 (1:200; Affinity, AF0133). The paraffin sections of mice kidney tumors and lung metastases were used to perform immunohistochemical staining to measure the protein levels of Cleaved-Caspase 3 (1:200; Affinity, AF7022), E-cadherin (1:500; Abcam, ab40772) and N-cadherin (1:1000; Abcam, ab19348), respectively.

### Immunofluorescence

OSRC2 and Caki-1 cells were seeded on laser confocal petri dish after transfection with the corresponding vector for 48 h. Sections were then incubated with primary antibodies against TOMM20 (1:500; Abcam, ab283317) and CLDN10 (1:500; Immunoway, YT0944) followed by incubation with the appropriate secondary antibody. Finally, the sections were visualized under a confocal laser microscope.

### Cell Apoptosis assays

One Step TUNEL Apoptosis Assay Kit (Beyotime, China) was used to detect cell apoptosis in cell slide. Colorimetric TUNEL Apoptosis Assay Kit (Beyotime, China) was used to detect cell apoptosis in the paraffin sections of mice kidney tumors.

### Flow cytometry assays

Cell apoptosis was also assayed by staining with Annexin V-FITC and PI (Beyotime, China), ROS level was assayed by staining with DCFH-DA (Beyotime, China) and MitoSOX (YEASEN, China). Finally, a flow cytometer was used to detect the fluorescence level of the cells.

### Cell proliferation assays

Cell proliferation was determined by an ethynyl-2-deoxyuridine (EdU) incorporation assay using an EdU Apollo DNA *in vitro* kit (RiboBio, Guangzhou, China) and BeyoClick™ EdU Cell Proliferation Kit with DAB (Beyotime, China). For cell clone formation experiment, 200 OSRC2 and Caki-1 cells were seeded in 6-well plates. After culturing for 2 to 3 weeks, stain with 0.5% crystal violet for 10 min and count the number of clones under a microscope. Experiments were repeated at least three times.

### Cell transwell migratory and invasive assays

For cell transwell migration assay, 2×10^3^ OSRC2 and Caki-1 cells were plated into the upper chambers (24-well insert, pore size 8 μm, Corning) with 100 μL serum-free DMEM. The lower chambers were filled with 600 μL DMEM containing 10% FBS. 48 h later, cells under the surface of the lower chamber were washed with PBS and stained with 0.5% crystal violet for 10 min. For cell invasion assay, 2×10^3^ cells were seeded on upper chambers coated with 100 μL Matrigel (1:8 dilution in PBS, Corning). The culture conditions were the same as described for the transwell migration assay. After 48 h, adherent cells on the lower surface were stained with 0.5% crystal violet. The number of cells on the lower surface was photographed with a microscope.

### Co-IP (Co-immunoprecipitation)

Co-IP assay was employed to analyze the interaction between CLDN10 and ATP5O protein, SIRT3 and ATP5O protein. Cells were lysed using IP lysis buffer (Beyotime, China). Anti-Flag M2 magnetic beads (Sigma, USA) were added into the supernatant of cell lysates and incubated overnight at 4 °C. SIRT3 antibodies were added into the supernatant of cell lysates and incubated overnight at 4 °C, and the subsequent incubation of complexes was done with protein A/G magnetic beads (Sigma, USA) for 2 h at room temperature. At last, the Co-IP products were harvested after being washed 3 times with lysis buffer and analyzed by silver-staining, mass spectrometry and western blot.

### TMT (Tandem mass tags) quantitative proteomics test

The TMT test is completed by the Shanghai Biotree Biomedical Technology company. The brief steps are as follows: for each sample, 2 ug of total peptides were separated and analyzed with a nano-UPLC (EASY-nLC1200) coupled to a Q Exactive HFX Orbitrap instrument (Thermo Fisher Scientific) with a nano-electrospray ion source. Separation was performed using a reversed-phase column (100 µID ×15 cm, Reprosil-Pur 120 C18-AQ, 1.9 µ, Dr. Maisch). Mobile phases were H2O with 0.1% FA, 2% ACN (phase A) and 80% ACN, 0.1% FA (phase B). Separation of sample was executed with a 90 min gradient at 300 nL/min flow rate. Gradient B: 2-5% for 2 min, 5-22% for 68 min, 22-45% for 16 min, 45-95% for 2 min, 95% for 2 min.

### Mitochondrial membrane potential detection

Mitochondrial membrane potential was detected using the enhanced mitochondrial membrane potential assay kit with JC-1 (Beyotime, China) according to the instructions.

### Deacetylation

NAD+ (Nicotinamide adenine dinucleotide, aladdin, N111610), a type III histone deacetylase sirtuins agonist, was used to inhibit the acetylation level of ATP5O, and the working concentration of NAD+ was 10 uM.

### Orthotopic tumor growth model

Six-week old B-NDG severe immunodeficiency mice (Biocytogen Pharmaceuticals (Beijing) Co., Ltd) were used for xenograft studies. Approximately 10×10^5^ viable Caki-1 cells were resuspended in 20μl fresh PBS and injected orthotopically into the right kidney of each mouse. Bioluminescence imaging was performed as described previously 27. EdU (50 mg/kg) was injected intraperitoneally 2 hours before the mice were sacrificed. After mice were sacrificed, lung *ex vivo* imaging was performed immediately to examine tumor metastasis. All procedures were approved by the Institutional Animal Care and Use Committee at Peking University First Hospital.

### Statistical analyses

Non-parametric Mann-Whitney test was used to detect differences in continuous variables. Survival curves for patients were plotted using the Kaplan-Meier method, with log-rank tests for statistical significance. The correlation of CLDN10 with ATP5O, NDUFS2, NDUFB8, SDHB, UQCRC2, MT-CO2 and ATP5A1 and the correlation of ATP5O with NDUFS2 mRNA expression in ccRCC were examined using Pearson's correlation analysis. All statistical tests were two-sided, and a *P* value of < 0.05 was regarded as statistical difference.

## Results

### Decreased CLDN10 protein expression is related to ccRCC metastasis and poor prognosis

**Figure 1:**
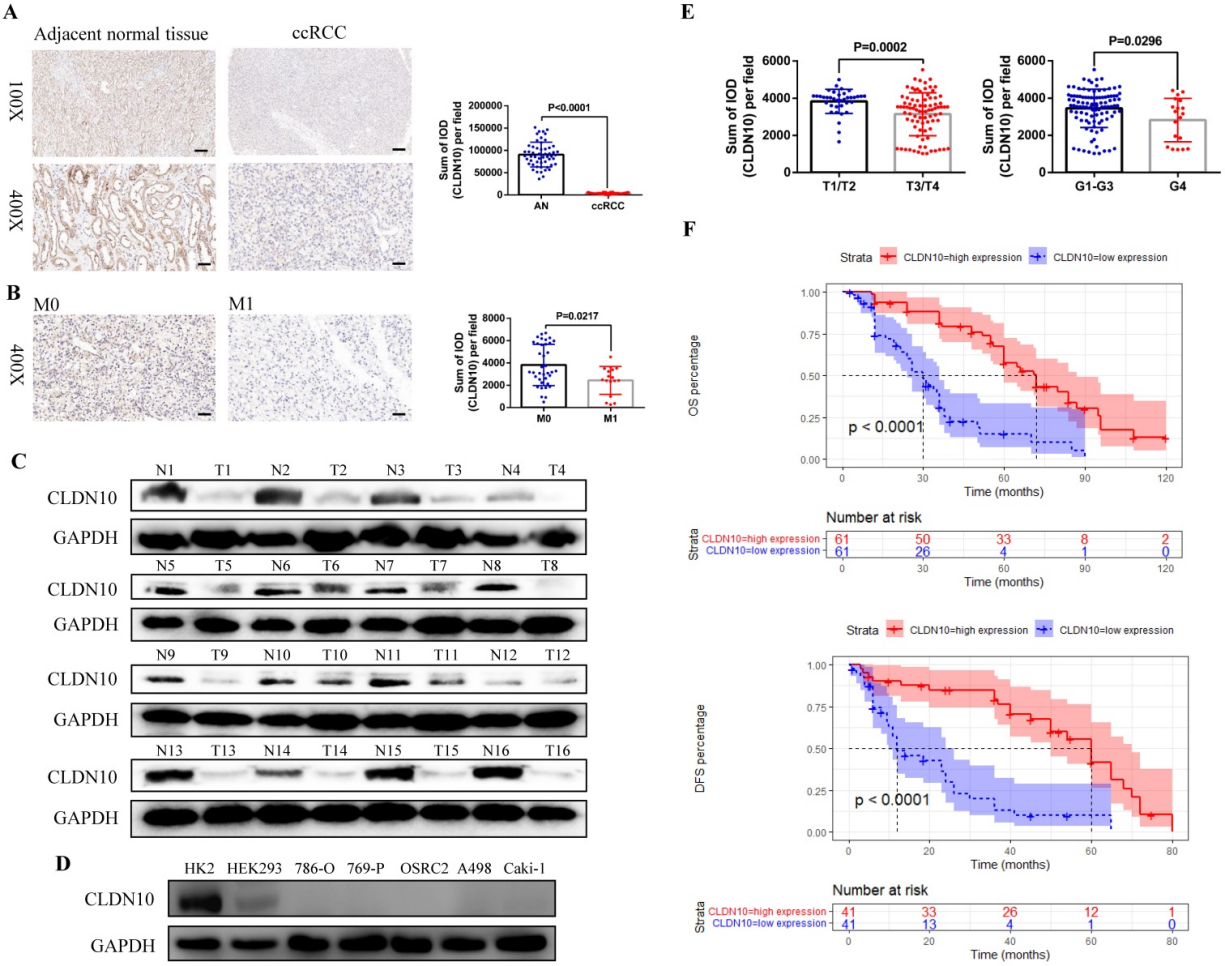
** CLDN10 is low expressed in ccRCC and decreased CLDN10 expression is associated with ccRCC metastasis and poor prognosis. A,** CLDN10 expression was analyzed by immunohistochemistry in 54 pairs of paraffin sections of ccRCC and adjacent normal renal tissues (AN). **B,** Comparison of CLDN10 expression in non-mccRCC (M0, n=37) and mccRCC (M1, n=17) in these 54 samples. Bar: 100*um* (100X), 25*um* (400X). **C,** CLDN10 expression was analyzed by western blot in 16 pairs of fresh mccRCC (T) and adjacent normal renal tissues (N). **D,** CLDN10 expression was analyzed by western blot in 5 ccRCC cell lines (786-O, 769-P, OSRC2, A498 and Caki-1) and 2 relative control cell lines (HK2 and HEK293). **E,** Comparison of CLDN10 expression in T1/T2 group (n=37) and T3/T4 group (n=85), G1-G3 group (n=103) and G4 group (n=19) in 122 mccRCC patients. **F,** Comparison of the prognostic differences between CLDN10 high expression and low expression groups in 122 mccRCC patients.

First, in order to clarify the expression status of CLDN10 protein in ccRCC, we used immunohistochemistry to examine CLDN10 expression in the paraffin sections of 54 pairs of ccRCC and adjacent normal renal tissues. Results showed that CLDN10 expression was significantly down-regulated in ccRCC compared to the adjacent normal renal tissues (**Fig. 1A**). Moreover, we found that CLDN10 was less expressed in mccRCC tissues by combining with the pathological information of these 54 specimens (**Fig. 1B**). To verify the expression of CLDN10 in mccRCC, western blot was utilized to examine CLDN10 expression in 16 pairs of fresh mccRCC and adjacent normal renal tissues, and results determined that CLDN10 expression was also remarkably reduced in mccRCC (**Fig. 1C**). Besides, we tested the expression of CLDN10 in five ccRCC cell lines (786-O, 769-P, OSRC2, A498 and Caki-1) and two control cell lines (HK2 and HEK293), and found that CLDN10 expression was generally reduced in these ccRCC cells compared to the HK2 cells (**Fig. 1D**).

In addition, we obtained the paraffin sections of 122 patients with mccRCC and their clinicopathological and prognostic information (**Table 1**), and detected the expression of CLDN10 in these 122 sections by immunohistochemistry. By combining the CLDN10 expression data and clinical information of these 122 samples, we found that decreased CLDN10 expression was related to advanced tumor stage (T3/T4) and higher tumor grade (G4) (**Fig. 1E**). Moreover, the Kaplan-Meier analysis results showed that the CLDN10 high expression group had longer OS and DFS compared to the CLDN10 low expression group (**Fig. 1F**). Furthermore, univariate and multivariate Cox regression analysis results determined that lower CLDN10 expression was an independent predictor of shorter OS (HR: 4.0860, 95%CI: 2.4737-6.7490, P<0.0001) (**Table 2**) and DFS (HR: 4.3680, 95%CI: 2.2800-8.3700, P<0.0001) (**Table 3**) in mccRCC patients.

### CLDN10 protein overexpression promotes cell apoptosis and suppresses cell proliferation, migration and invasion *in vitro*

**Figure 2:**
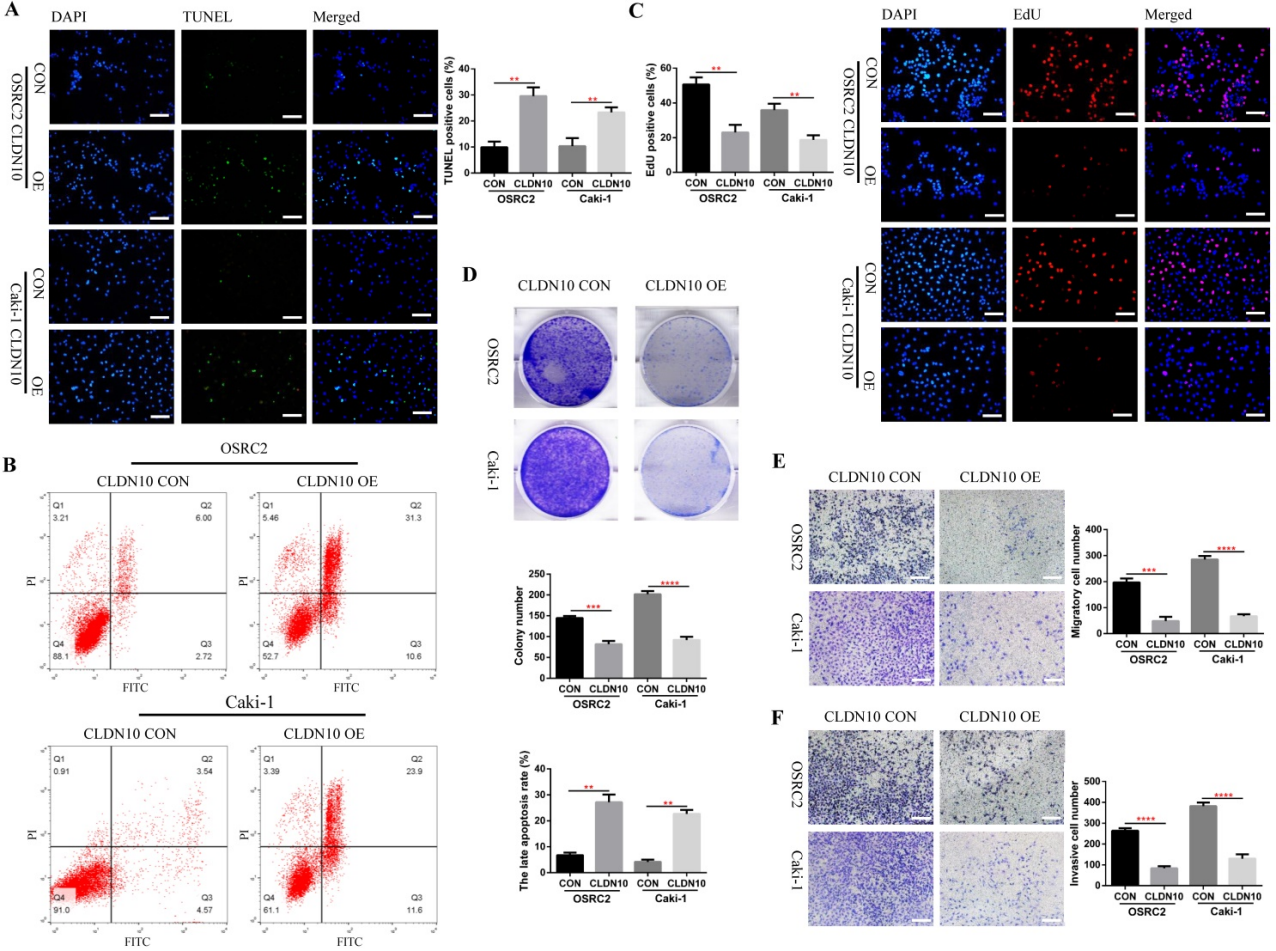
** CLDN10 overexpression promotes cells apoptosis and inhibits cells proliferation, migration and invasion *in vitro*. A,** Comparison of the proportion of TUNEL positive cells in the CLDN10 overexpression and the control Caki-1 and OSRC2 cells by immunofluorescence (200X, Bar: 50*um*). **B,** Comparison of the proportion of apoptotic cells in the CLDN10 overexpression and the control Caki-1 and OSRC2 cells by flow cytometry. **C,** Comparison of the proportion of EdU positive cells in the CLDN10 overexpression and the control Caki-1 and OSRC2 cells by immunofluorescence (200X, Bar: 50*um*). **D,** Comparison of the number of cell clones in the CLDN10 overexpression and the control Caki-1 and OSRC2 cells. Comparison of cell migration (**E**) and cell invasion (**F**) ability in the CLDN10 overexpression and the control Caki-1 and OSRC2 cells (100X, Bar: 100*um*). ^**^P < 0.01, ^***^P < 0.001, ^****^P < 0.0001.

Now that we have fully determined the low expression status of CLDN10 protein in ccRCC, does the overexpression of CLDN10 affect the phenotypes of ccRCC cells? To this end, we constructed CLDN10 stably transfected OSRC2 and Caki-1 cell lines. The effect of changes in CLDN10 expression on the apoptosis of ccRCC cells was detected by the immunofluorescence TUNEL method and flow cytometry, the effect of changes in CLDN10 expression on the growth of ccRCC cells was detected by the immunofluorescence EdU method and clone formation experiment, and the effect of changes in CLDN10 expression on the metastatic ability of ccRCC cells was detected by cell migration and invasion experiments. Our results suggested that CLDN10 overexpression significantly increased apoptosis rate in OSRC2 and Caki-1 cells (**Fig. 2A and B**) and inhibited the ability of growth (**Fig. 2C and D**), migration (**Fig. 2E**), and invasion (**Fig. 2F**) in OSRC2 and Caki-1 cells.

### CLDN10 protein binds to ATP5O protein and increases its expression

**Figure 3:**
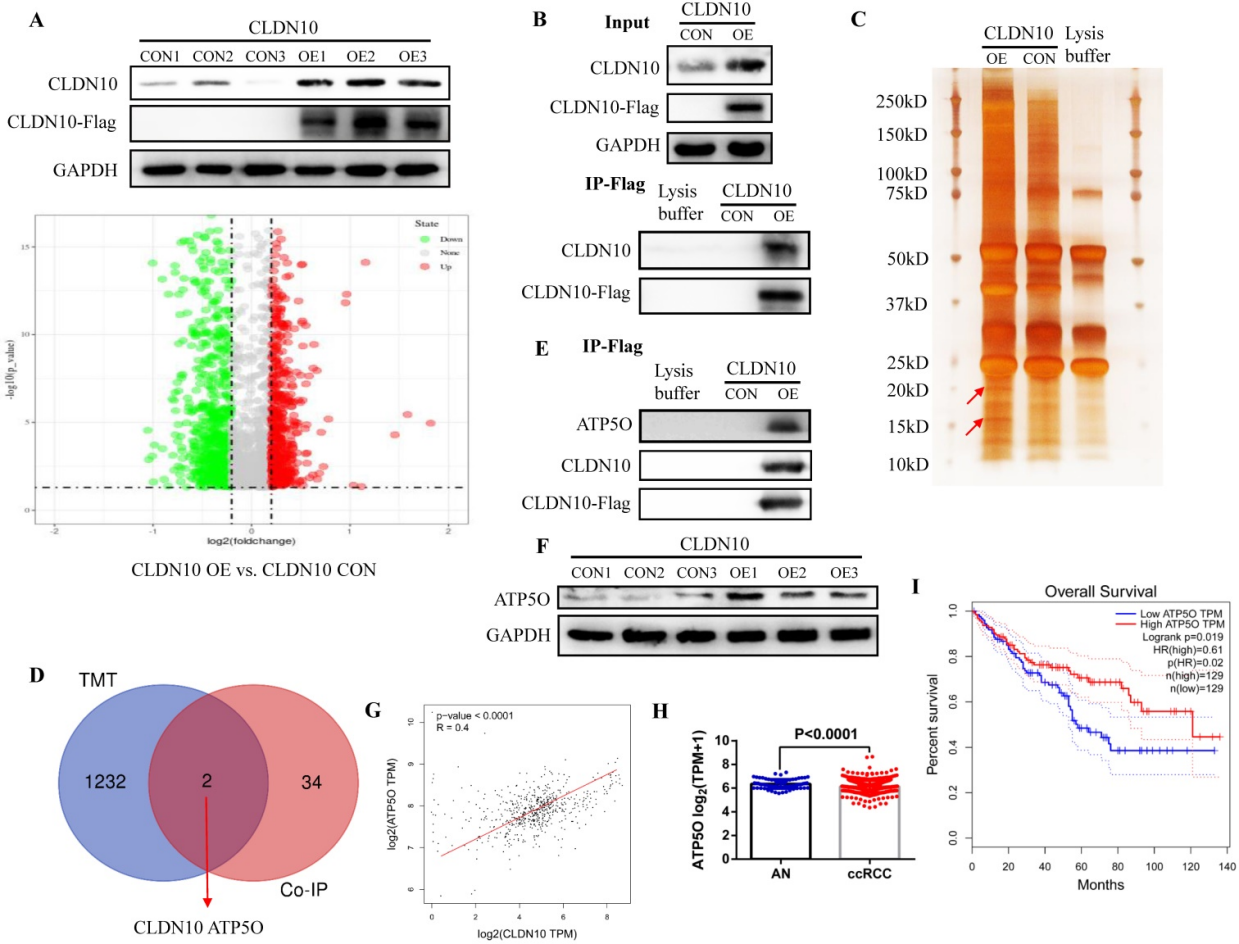
** CLDN10 protein binds to ATP5O protein and increases its expression. A,** Comparison of the differential protein between CLDN10 overexpression and the control Caki-1 cells by TMT experiment. Identification of the possible binding proteins of CLDN10 protein by IP (**B**), silver-staining and mass spectrometry (**C**). **D,** Venn diagram analysis of TMT results and mass spectrometry results. **E,** Confirmation of ATP5O protein binding to CLDN10 protein by western blot. **F,** Comparison of ATP5O expression between CLDN10 overexpression and the control Caki-1 cells. The correlation between ATP5O expression and CLDN10 expression (n=539) (**G**), the comparison of ATP5O expression in ccRCC (n=539) and adjacent normal tissues (AN, n=72) (**H**), and the relationship between ATP5O expression and the prognosis of ccRCC patients (n=539) (**I**) were analyzed using TCGA-KIRC data.

In order to explore the mechanism of CLDN10 overexpression inhibiting the phenotype of ccRCC cells, TMT method was used to detect the differentially expressed proteins in the CLDN10 overexpressed Caki-1 cells and its control Caki-1 cells. TMT results indicated that compared with the control group, 564 proteins and 670 proteins were up-regulated and down-regulated in the CLDN10 overexpressed Caki-1 cells, respectively (**Fig. 3A**). Besides, Co-IP experiment was utilized to identify proteins that may bind to CLDN10. In the input control samples, CLDN10 expression was significantly increased in CLDN10 overexpression group, and Flag could only be detected in CLDN10 overexpression group; in the IP samples, both CLDN10 and Flag could only be detected in CLDN10 overexpression group (**Fig. 3B**). Silver-staining method was used to detect the differential protein bands between CLDN10 overexpression group and its control group, and we can clearly identify two different protein bands in CLDN10 overexpression group (**Fig. 3C**). Then, the two different protein bands were sent to mass spectrometry test to detect possible proteins in them, and results suggested that 36 proteins were detected. By combining the TMT data and mass spectrometry data, we speculated that ATP5O may be the binding protein of CLDN10, and its expression was regulated by CLDN10 (**Fig. 3D**).

Consistent with the hypothesis, in the same IP protein samples, ATP5O protein could only be detected in CLDN10 overexpression group (**Fig. 3E**). Besides, we also examined the expression of ATP5O in CLDN10 overexpression group and its control group, the results verified that ATP5O expression was remarkably increased in CLDN10 overexpression group (**Fig. 3F**). In addition, we used TCGA-KIRC data to analyze the correlation between CLDN10 expression and ATP5O expression, and the results confirmed that CLDN10 expression was significantly positively correlated with ATP5O expression (**Fig. 3G**). Further, we also found ATP5O expression was remarkably reduced in ccRCC (**Fig. 3H**), and higher ATP5O expression was associated with a favorable prognosis (**Fig. 3I**). Based on these results, we confirmed that CLDN10 protein could bind to ATP5O protein and cause its expression up-regulation.

### CLDN10 locates in mitochondria and promotes the acetylation modification of ATP5O protein

**Figure 4:**
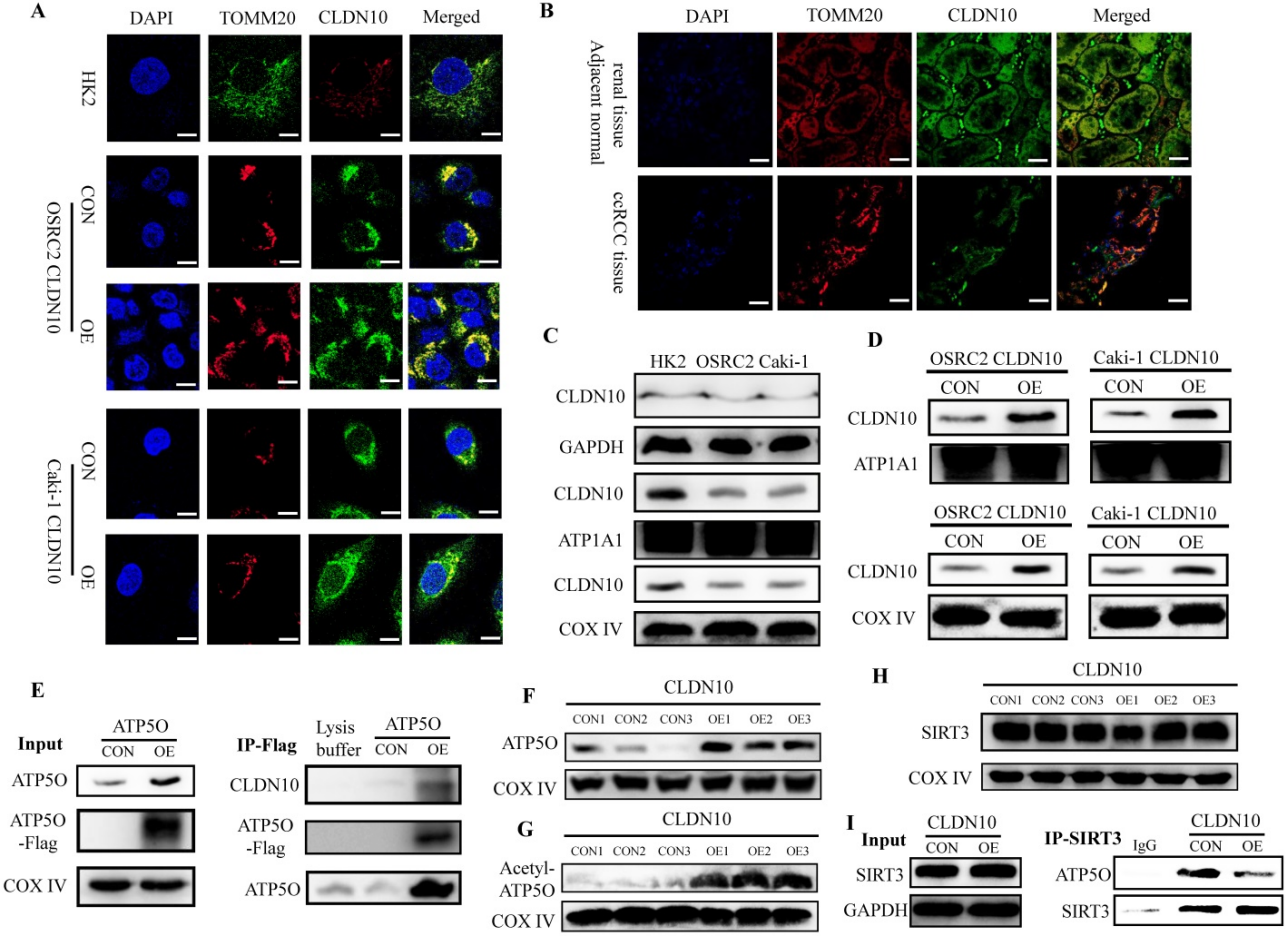
** CLDN10 locates in mitochondria and promotes the acetylation modification of ATP5O protein.** Clarification of the sub-localization of CLDN10 in HK2, OSRC2 and Caki-1 cells (**A**), and ccRCC and adjacent normal renal tissues (**B**), TOMM20 as a marker for the mitochondrial outer membrane (600X, Bar: 15*um*). **C,** Comparison of the expression of CLDN10 in the cytoplasm, cell membrane and mitochondria of HK2, OSRC2 and Caki-1 cells. **D,** Comparison of the expression of CLDN10 in the cell membrane and mitochondria of CLDN10 overexpression OSRC2 and Caki-1 cells. **E,** Identification of the binding of ATP5O and CLDN10 protein in the mitochondria of Caki-1 cells. **F,** Comparison of ATP5O expression in the mitochondria of CLDN10 overexpression and the control Caki-1 cells. **G,** Comparison of the expression level of acetylated ATP5O protein in the mitochondria of CLDN10 overexpression and the control Caki-1 cells. **H,** Comparison of SIRT3 expression in the mitochondria of CLDN10 overexpression and the control Caki-1 cells. **I,** Comparison of the content of ATP5O protein combined with SIRT3 in the mitochondria of CLDN10 overexpression and the control Caki-1 cells.

ATP5O protein (ATP synthase subunit O, mitochondrial) is a component of the F-type ATPase found in the mitochondrial matrix, and CLDN10 is generally considered to be located on the cell membrane. Therefore, in order to further determine the interaction between CLDN10 and ATP5O, we used immunofluorescence and confocal laser microscopy to clarify the sub-localization of CLDN10 in cells. Results indicated that the expression of CLDN10 and TOMM20 (a mitochondrial marker located in mitochondrion outer membrane) have a consistent location in HK2, OSRC2 and Caki-1 cells (**Fig. 4A**). We also detected the sub-localization of CLDN10 expression in ccRCC and adjacent normal renal tissues, and obtained the same results as in cells (**Fig. 4B**). Besides, we separately detected the CLDN10 protein expression in cell cytoplasm, membrane and mitochondrion by western blot, and GAPDH, ATP1A1, COX Ⅳ were used as control proteins for cytoplasm, cell membrane and mitochondrial proteins, respectively. Results indicated that CLDN10 protein existed in cell membrane and mitochondrion (**Fig. 4C**). Moreover, after CLDN10 overexpression, the level of CLDN10 protein in cell membrane and mitochondrion was significantly increased (**Fig. 4D**). Moreover, we constructed an ATP5O overexpression plasmid (with 3xflag) and screened out ATP5O stably transfected Caki-1 cell line. The results of IP experiment using the mitochondrial protein also found that ATP5O protein could bind to CLDN10 protein (**Fig. 4E**).

Additionally, we examined the expression of ATP5O in the mitochondria of CLDN10 overexpression Caki-1 cells and its control cells, and results also indicated that ATP5O expression was also remarkably increased in the mitochondria of CLDN10 overexpressed Caki-1 cells (**Fig. 4F**). Interestingly, we found that the expression of acetylated ATP5O protein was also up-regulated in the mitochondrial of CLDN10 overexpressed Caki-1 cells (**Fig. 4G**). In order to find out whether it was the change of ATP5O deacetylase that caused the change of ATP5O acetylation modification level, we examined the expression of SIRT3 (NAD-dependent protein deacetylase sirtuin-3, mitochondrial), which can deacetylate ATP5O 20. However, the results showed that overexpression of CLDN10 did not cause changes in the expression of SIRT3 (**Fig. 4H**). Thus, we speculated that although CLDN10 did not cause alteration in the expression of SIRT3, it may affect the binding of SIRT3 and ATP5O. Consistent with the speculated result, the IP experiment result confirmed that the ATP5O protein binding to SIRT3 was significantly reduced in the CLDN10 overexpression group compared to the control group (**Fig. 4I**). Taken together, our results suggested that CLDN10 could locate in the mitochondria of ccRCC cells and effected the acetylation modification of ATP5O protein.

### CLDN10 overexpression increases NDUFS2 expression by regulating ATP5O

**Figure 5:**
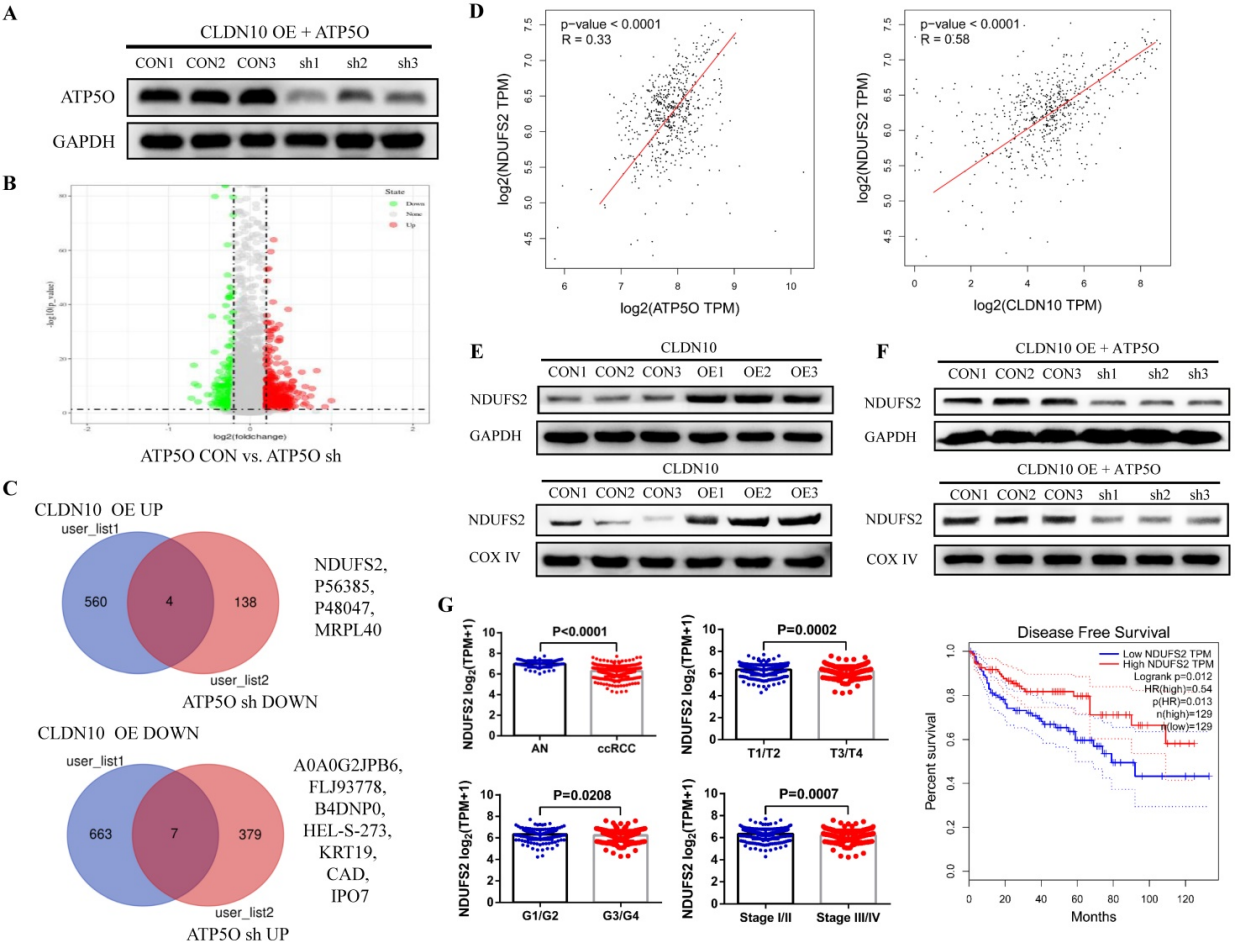
** CLDN10 overexpression increases NDUFS2 expression by regulating ATP5O. A,** The expression of ATP5O was knocked down in CLDN10 overexpression Caki-1 cells. **B,** Comparison of the differential protein between ATP5O knockdown and its control Caki-1 cells by TMT experiment. **C,** Venn diagram analysis of the two TMT test data. **D,** The correlation between ATP5O and NDUFS2 expression, and CLDN10 and DNUFS2 expression were analyzed using TCGA-KIRC data (n=539). **E,** Comparison of the expression of NDUFS2 in the total protein and mitochondrial protein of CLDN10 overexpression and the control Caki-1 cells. **F,** Comparison of the expression of NDUFS2 in the total protein and mitochondrial protein of ATP5O knockdown and its control Caki-1 cells. **G,** The comparison of NDUFS2 expression in ccRCC (n=539) and adjacent normal renal tissues (AN, n=72), T1/T2 group (n=340) and T3/T4 group (n=190), G1/G2 group (n=241) and G3/G4 group (n=281) and Stage I/II group (n=322) and Stage III/IV group (n=205) and the relationship between NDUFS2 expression and the prognosis of ccRCC patients were analyzed using TCGA-KIRC data.

Currently, the role of ATP5O in tumors is still very vague. To explore the mechanism of ATP5O in ccRCC, we knocked down the expression of ATP5O on the basis of overexpression of CLDN10 in Caki-1 cells (**Fig. 5A**). TMT results showed that compared with the control group, 386 proteins and 142 proteins were up-regulated and down-regulated in the ATP5O sh group, respectively (**Fig. 5B**). Combining the previous TMT data of CLDN10 overexpression and its control group, we found 4 proteins (NDUFS2, P56385, P48047, MRPL40) were up-regulated in CLDN10 overexpression group and down-regulated in ATP5O knockdown group, and 7 proteins (A0A0G2JPB6, FLJ93778, B4DNP0, HEL-S-273, KRT19, CAD, IPO7) were down-regulated in CLDN10 overexpression group and up-regulated in ATP5O knockdown group (**Fig. 5C**). Among these 11 proteins, NDUFS2 (NADH dehydrogenase [ubiquinone] iron-sulfur protein 2, mitochondrion inner membrane) expression was significantly positively correlated with the expression of CLDN10 and ATP5O based on TCGA-KIRC data (**Fig. 5D**). Moreover, our western blot results confirmed that NDUFS2 protein expression was remarkably increased in the total protein and mitochondrial protein of CLDN10 overexpression group (**Fig. 5E**) and decreased in the total protein and mitochondrial protein of ATP5O knockdown group (**Fig. 5F**). In addition, using the TCGA-KIRC data, we found that DNUFS2 expression was decreased in ccRCC, and lower NDUFS2 expression was associated with worse pathological characteristics, including T3/T4, G3/G4 and Stage III/IV, and predicted a poor prognosis of ccRCC patients (**Fig. 5G**).

**Figure 6:**
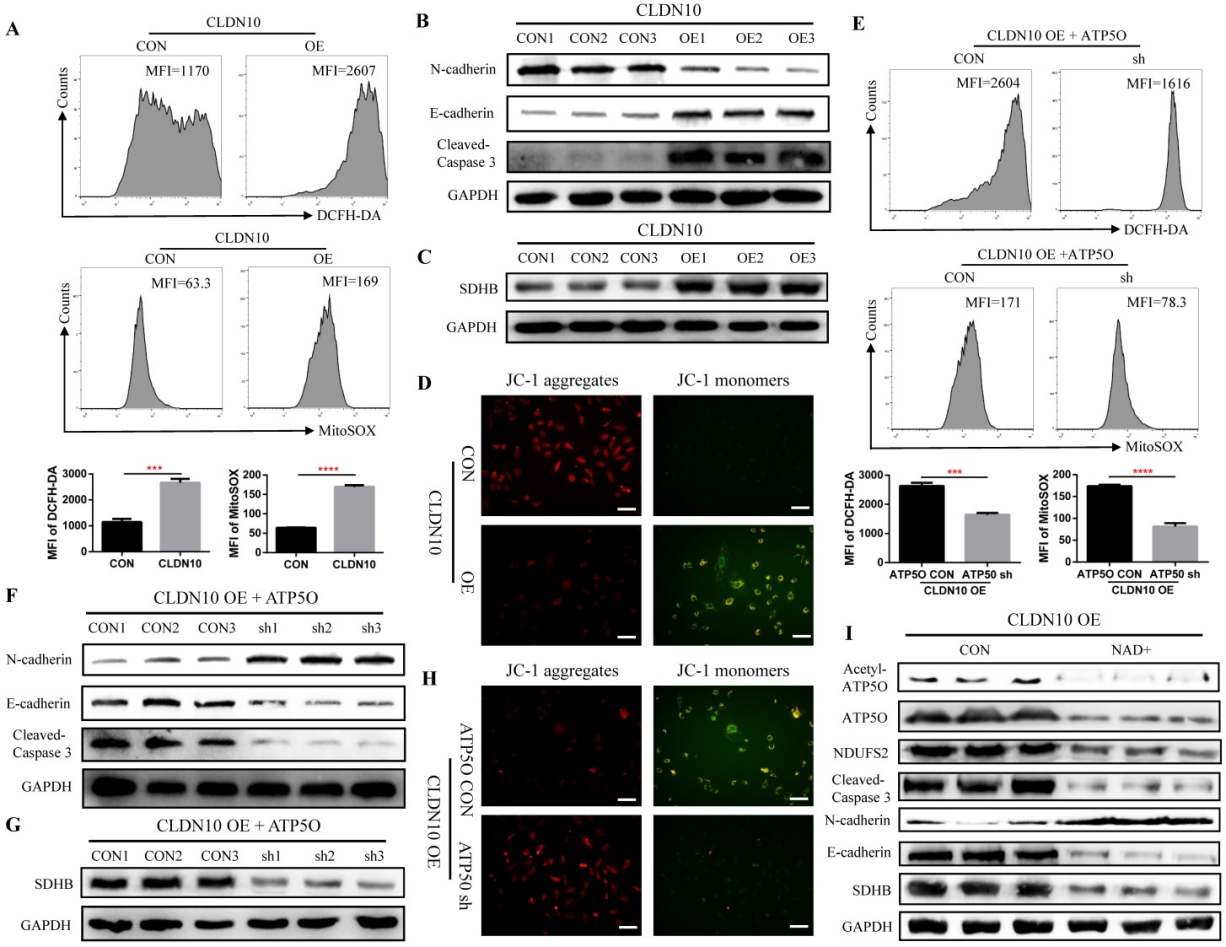
** CLDN10 overexpression increases the levels of ROS, Cleaved-Caspase 3, E-cadherin and SDHB and decreases the levels of N-cadherin and mitochondrial membrane potential. A,** Comparison of the ROS levels in CLDN10 overexpression and the control Caki-1 cells. **B,** Comparison of the expression of Cleaved-Caspase 3, E-cadherin and N-cadherin in CLDN10 overexpression and the control Caki-1 cells. **C,** Comparison of the expression of SDHB in CLDN10 overexpression and the control Caki-1 cells. **D,** Comparison of mitochondrial membrane potential in CLDN10 overexpression and the control Caki-1 cells (when the mitochondrial membrane potential is high, JC-1 aggregates in the matrix of mitochondria and forms JC-1 aggregates, which produces red fluorescence; when the mitochondrial membrane potential is low, JC-1 cannot aggregate in the matrix of mitochondria, at this time, JC-1 is a monomer and can produce green fluorescence). **E,** Comparison of the ROS levels in ATP5O knockdown and its control Caki-1 cells. **F,** Comparison of the expression of Cleaved-Caspase 3, E-cadherin and N-cadherin in ATP5O knockdown and its control Caki-1 cells. **G,** Comparison of the expression of SDHB in ATP5O knockdown and its control Caki-1 cells. **H,** Comparison of mitochondrial membrane potential in ATP5O knockdown and its control Caki-1 cells. **I,** The effects of NAD+ inhibiting ATP5O acetylation on the expression of ATP5O, NDUFS2, Cleaved-Caspase 3, N-cadherin, E-cadherin and SDHB. ^***^P < 0.001, ^****^P < 0.0001.

As a core subunit of the mitochondrial membrane respiratory chain NADH dehydrogenase (Complex I), NDUFS2 is thought to be involved in ROS (reactive oxygen species) generation, OXPHOS (oxidative phosphorylation), and cell apoptosis 28-30. First, we detected the level of ROS by flow cytometry, and results indicated that the ROS level in CLDN10 overexpression group was significant increased (**Fig. 6A**). The protein expression of Cleaved-Caspase 3 and E-cadherin was also significantly increased and the protein expression of N-cadherin was significant decreased in CLDN10 overexpression group compared to its control group (**Fig. 6B**). The correlation of CLDN10 expression with the expression of five key OXPHOS complexes (NDUFB8, SDHB, UQCRC2, MT-CO2, ATP5A1) was also analyzed using the TCGA-KIRC data, and results indicated that the expression of CLDN10 was significantly correlated with these five genes expression, and the correlation with SDHB was the strongest (Supplementary Figure 1A). Besides, increased SDHB expression was associated with better prognosis in ccRCC patients (Supplementary Figure 1B). Our western blot results also verified that the expression of SDHB was remarkably increased after CLDN10 overexpression (Fig. 6C). In addition, we examined the changes of mitochondrial membrane potential after CLDN10 overexpression, and found that the mitochondrial membrane potential level was significantly decreased in CLDN10 overexpression cells (Fig. 6D). Moreover, we confirmed that knocking down the expression of ATP5O based on the overexpression of CLDN10 could decrease the levels of ROS, Cleaved-Caspase 3, E-cadherin and SDHB and increase the level of N-cadherin and mitochondrial membrane potential (**Fig. 6E, F, G and H**).

To further demonstrate whether CLDN10 affects the expression of NDUFS2 and its downstream pathways by regulating the acetylation of ATP5O, we used NAD+ to inhibit the acetylation of ATP5O based on CLDN10 overexpression. Our results confirmed that NAD+ could significantly inhibit the acetylation level of ATP5O and its expression level, while the expression of NDUFS2, Cleaved-Caspase 3, E-cadherin and SDHB were decreased and the expression of N-cadherin was increased (Fig. 6I). Based on the above results, we concluded that CLDN10 overexpression increased NDUFS2 expression by regulating the acetylation of ATP5O, and enhanced NDUFS2 expression could cause mitochondrial dysfunction.

### Knockdown of ATP5O expression reverses the phenotype inhibition of ccRCC cells caused by CLDN10 overexpression *in vitro* and* in vivo*

**Figure 7:**
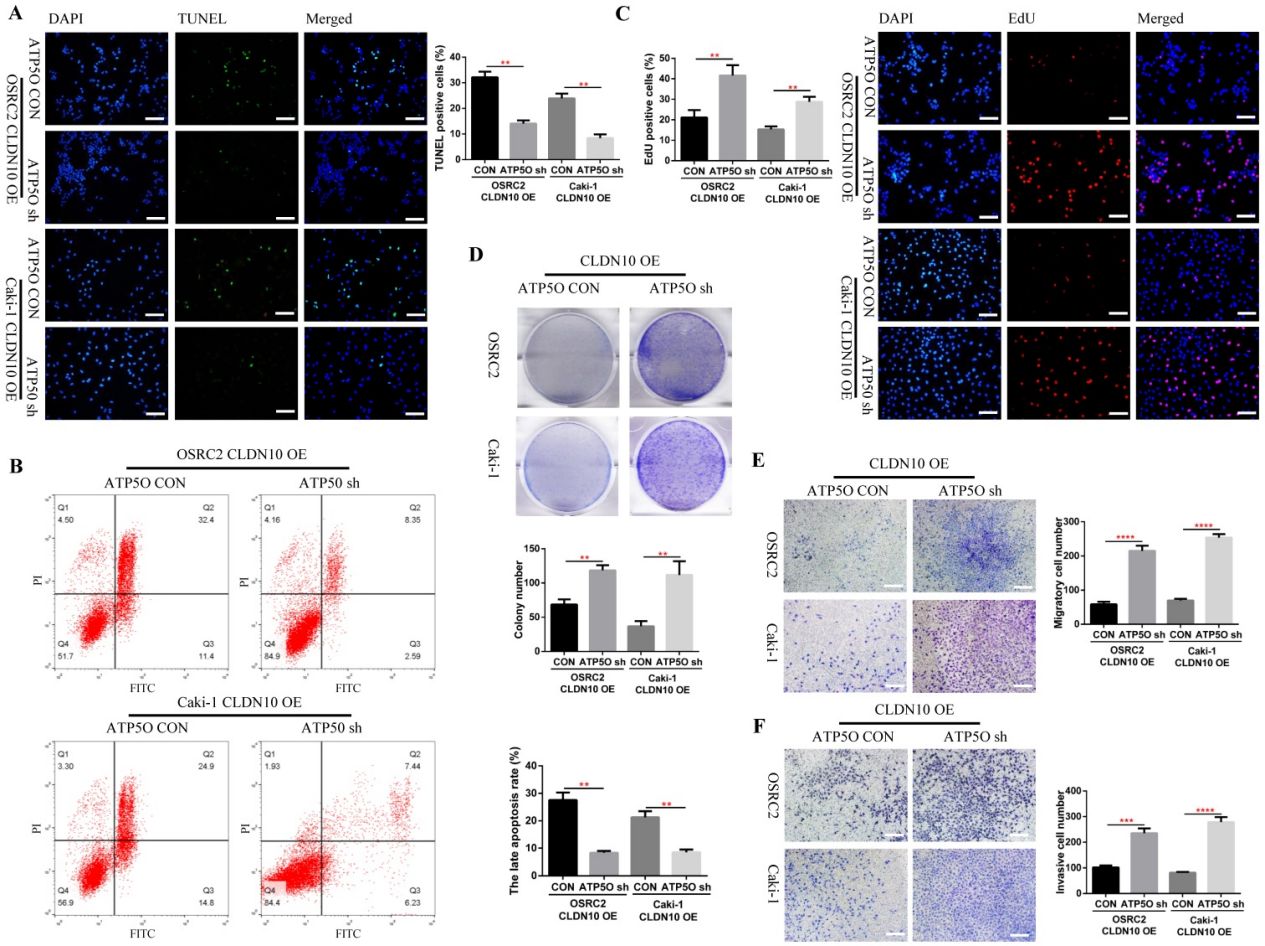
** Knockdown of ATP5O expression reverses the phenotype inhibition of ccRCC cells caused by CLDN10 overexpression *in vitro*. A,** Comparison of the proportion of TUNEL positive cells in ATP5O knockdown and its control Caki-1 and OSRC2 cells by immunofluorescence (200X, Bar: 50*um*). **B,** Comparison of the proportion of apoptotic cells in ATP5O knockdown and its control Caki-1 and OSRC2 cells by flow cytometry. **C,** Comparison of the proportion of EdU positive cells in ATP5O knockdown and its control Caki-1 and OSRC2 cells by immunofluorescence (200X, Bar: 50*um*). **D,** Comparison of the number of cell clones in ATP5O knockdown and its control Caki-1 and OSRC2 cells. Comparison of cell migration (**E**) and cell invasion (**F**) ability in ATP5O knockdown and its control Caki-1 and OSRC2 cells (100X, Bar: 100*um*). ^**^P < 0.01, ^***^P < 0.001, ^****^P < 0.0001.

Considering that knockdown of ATP5O could reverse the increase of NDUFS2, ROS, Cleaved-Caspase 3, E-cadherin and SDHB levels and the decrease of N-cadherin and mitochondrial membrane potential levels caused by CLDN10 overexpression, the question is whether knockdown of ATP5Oexpression could also reverse the increased apoptosis and inhibited cell proliferation, migration and invasion ability caused by CLDN10 overexpression. Our results confirmed that after knocking down the expression of ATP5O based on the overexpression of CLDN10, the cell apoptosis rate was significantly decreased (**Fig. 7A and B**) and the cell proliferation (**Fig. 7C and D**), migration and invasion ability (**Fig 7E and F**) was remarkably enhanced.

**Figure 8:**
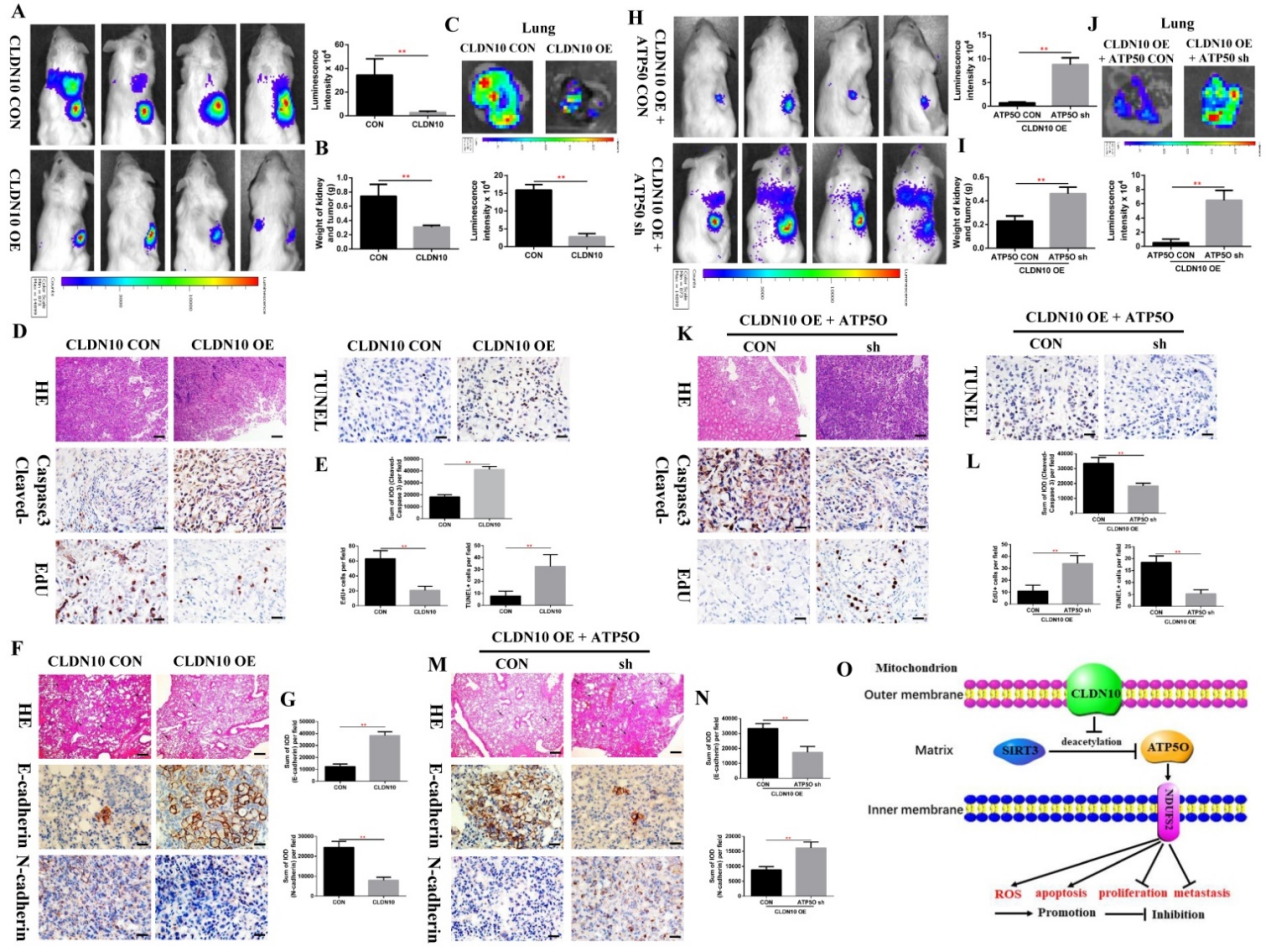
** CLDN10 overexpression inhibits ccRCC cells growth and metastasis *in vivo*, while this inhibition is obviously weakened after ATP5O knockdown. A,** Representative bioluminescence imaging of CLDN10 overexpression and the control groups and quantification of these bioluminescence imaging. **B,** Quantification of kidney and tumor weight of CLDN10 overexpression and the control groups. **C,** Representative lung *ex vivo* bioluminescence imaging of CLDN10 overexpression and the control groups and quantification of *ex vivo* imaging. Representative picture of HE (100X, Bar: 100*um*), Cleaved-Caspase 3, EdU and TUNEL expression (400X, Bar: 25*um*) in the kidney tumors of CLDN10 overexpression and the control groups (**D**) and quantification of these picture (**E**). Representative picture of HE (100X, Bar: 100*um*), E-cadherin and N-cadherin expression (400X, Bar: 25*um*) in the lung metastases of CLDN10 overexpression and the control groups (**F**) and quantification of these picture (**G**). **H,** Representative bioluminescence imaging of ATP5O knockdown and its control groups and quantification of these bioluminescence imaging. **I,** Quantification of kidney and tumor weight of ATP5O knockdown and its control groups. **J,** Representative lung *ex vivo* bioluminescence imaging of ATP5O knockdown and its control groups and quantification of *ex vivo* imaging. Representative picture of HE (100X, Bar: 100*um*), Cleaved-Caspase 3, EdU and TUNEL expression (400X, Bar: 25*um*) in the kidney tumors of ATP5O knockdown and its control groups (K) and quantification of these picture (**L**). Representative picture of HE (100X, Bar: 100*um*), E-cadherin and N-cadherin expression (400X, Bar: 25*um*) in the lung metastases of ATP5O knockdown and its control groups (**M**) and quantification of these picture (**N**). **O**, Schematic model of CLDN10 overexpression up-regulates the acetylation and expression levels of ATP5O protein, which leads to increase of NDUFS2 protein therefore suppressing the growth and metastasis of ccRCC. ^**^P < 0.01.

To verify the inhibitory effect of CLDN10 on ccRCC tumor growth and metastasis *in vivo*, we constructed a mouse orthotopic tumor growth model. Either CLDN10 overexpression or the control Caki-1 cells were orthotopically injected into the renal capsules of B-NDG severe immunodeficiency mice. Upon confirmation of tumor growth *in vivo* by bioluminescence imaging. Our results showed that the growth rate of CLDN10 overexpression cells was significantly slower than that in the control cells *in vivo* (**Fig. 8A and B**). Lung *ex vivo* imaging data indicated that CLDN10 overexpression also inhibited tumor cells' spontaneous lung metastasis (**Fig. 8C**). Besides, immunohistochemistry was used to detect the expression of EdU, TUNEL and Cleaved-Caspase 3 in kidney tumors and to detect the expression of E-cadherin and N-cadherin in lung metastases, results showed that the expression of EdU and N-cadherin were significantly decreased and the expression of TUNEL, Cleaved-Caspase 3 and E-cadherin were significantly increased in the kidney tumors and lung metastases of CLDN10 overexpression group (**Fig. 8D-G**). In addition, after knocking down the expression of ATP5O based on the overexpression of CLDN10, the tumor growth and spontaneous lung metastasis rate was significantly restored (**Fig. 8H-J**). Moreover, the increased TUNEL, Cleaved-Caspase 3 and E-cadherin expression and the decreased EdU and N-cadherin expression were remarkably reversed after knocking down the expression of ATP5O based on the overexpression of CLDN10 (**Fig. 8K-N**).

Taken together, our results provide novel mechanistic insight by which CLDN10 overexpression up-regulates the acetylation and expression levels of ATP5O protein, which leads to up-regulation of NDUFS2 protein, therefore suppressing the growth and metastasis of ccRCC by causing mitochondrial dysfunction (**Fig. 8O**).

## Discussion

In the past decade, many studies have emphasized the roles of claudins not only as prognostic markers of primary tumors, but also as functional effectors of the metastasis process. Currently, CLDN1-4, CLDN6, CLDN7 and CLDN9 are considered to be closely related to tumor metastasis, including lung cancer, prostate cancer, ovarian cancer, gastric cancer, colorectal cancer and breast cancer 31, 32. In this study, we examined CLDN10 expression in a large number of clinical ccRCC and mccRCC samples, and determined that CLDN10 expression was generally lost in kidney cancer. Besides, combining the clinicopathological and prognostic information of patients, we determined that CLDN10 expression was extremely low in mccRCC, and reduced CLDN10 expression was an independent predictor of poor prognosis. Moreover, through a large number of *in vitro* and *in vivo* experiments, we found that CLDN10 overexpression significantly inhibited the growth and metastasis of ccRCC cells. Based on these results, we identify that CLDN10 can not only predict the prognosis of ccRCC patients but also participate in the process of tumor growth and metastasis.

To clarify the mechanism of CLDN10 suppressing ccRCC growth and metastasis, we found that ATP5O was the binding protein of CLDN10 through TMT and IP experiments. It has been determined that ATP5O is located in the mitochondrial matrix, and claudins are generally believed to be present on the cell membrane 33, 34. Previous study has pointed out that CLDN5 was present in the mitochondria in cardiomyocytes 35. Now that we have identified that CLDN10 can bind to ATP5O, does CLDN10 also exist in mitochondria? Therefore, we used immunofluorescence to detect the sub-localization of CLDN10 and western blot to examine the expression of CLDN10 in cell membrane and mitochondrial. Both immunofluorescence and western blot results indicated that CLDN10 was not only expressed on the cell membrane but also on the outer mitochondrial membrane of ccRCC cells. Besides, we found that CLDN10 could not only bind to ATP5O but also up-regulate its expression. Previous study has showed that ATP5O protein had an acetylated form 36, and acetylation as an important form of post-translational modification can epigenetically regulate various properties of proteins 37. Thus, we examined the acetylation level of ATP5O, and results indicated that the expression of acetylated ATP5O protein was significantly increased in the CLDN10 overexpressed Caki-1 cells. In addition, we also examined the expression of SIRT3 protein (the deacetylase of ATP5O 21). However, the results showed that there was no difference in SIRT3 expression between CLDN10 overexpressed Caki-1 cells and its control cells, but we found that the level of ATP5O protein binding to SIRT3 protein was significantly decreased after CLDN10 overexpression. Thus, the above results confirm that CLDN10 overexpression can increase the acetylation level of ATP5O by affecting the binding of SIRT3 and ATP5O, thereby increasing its expression.

Now that we have shown that CLDN10 overexpression can up-regulate the expression of ATP5O, does the alteration of ATP5O expression cause changes in mitochondrial proteins expression and mitochondrial dysfunction? For this purpose, we knocked down the expression of ATP5O based on the overexpression of CLDN10 in Caki-1 cells, and used the TMT method to detect the differential proteins. Combining these two TMT test data, we screened out that NDUFS2 may be a mitochondrial protein regulated by ATP5O. Our western blot results indicated that NDUFS2 expression was up-regulated after CLDN10 overexpression and down-regulated after knocking down ATP5O expression based on the overexpression of CLDN10. Previous studies have suggested that NDUFS2 is related to the production of ROS, OXPHOS and the invasion ability of cancer 28, 30, 38. By analyzing the TCGA-KIRC data, we also found that DNUFS2 expression was decreased in ccRCC, and lower NDUFS2 expression was associated with worse pathology and poor prognosis. Besides, we found that the levels of ROS, Cleaved-Caspase 3, E-cadherin and SDHB were significant increased and the levels of N-cadherin and mitochondrial membrane potential were significant decreased after CLDN10 overexpression. Moreover, knocking down the expression of ATP5O based on the overexpression of CLDN10 could reverse the increase of ROS, Cleaved-Caspase 3, E-cadherin and SDHB levels and the decrease of N-cadherin and mitochondrial membrane potential levels caused by CLDN10 overexpression.

Although the above results have demonstrated that CLDN10 could induce changes in the expression NDUFS2 and its downstream pathways by regulating ATP5O expression, the role of changes in ATP5O acetylation in this process has not been fully elucidated. Thus, we used NAD+ to inhibit the acetylation level of ATP5O based on CLDN10 overexpression to further determine whether CLDN10 affects the expression of NDUFS2 and its downstream pathways by regulating the acetylation of ATP5O. Our results identified that the acetylation level of ATP5O and its expression level was significantly inhibited after NAD+ treatment based on CLDN10 overexpression, while the expression of NDUFS2, Cleaved-Caspase 3, E-cadherin and SDHB were decreased and the expression of N-cadherin was increased (Fig. 6I). Furthermore, knockdown of ATP5O expression could restore the suppression of ccRCC cell phenotype caused by CLDN10 overexpression *in vitro* and *in vivo*. Taken together, we confirm that CLDN10 overexpression up-regulates the acetylation and expression levels of ATP5O protein, which leads to the increase of NDUFS2 protein, therefore suppressing the growth and metastasis of ccRCC cells by causing mitochondrial dysfunction.

Loss or mutation of the von Hippel Lindau (*VHL*) gene is generally considered to be one of the most important causes of the occurrence and development of ccRCC. Some recent evidence suggested that *VHL* depletion led to several distinct patterns in cancer biology 39, 40. Previous studies have also pointed out that loss of VHL function significantly affected the expression of TJs component CLDN1 in *VHL*-deficient ccRCC cells *in vitro* and in *VHL*-deficient sporadic ccRCC *in vivo* 41. In this study, our results showed that the expression pattern of CLDN10 in *VHL*-depleted cell lines, especially in 786-O cells, was similar to that in other cell lines. However, there are currently few studies on the difference in the role of CLDN10 in *VHL*-deficient and *VHL* wild-type ccRCC cells, and this will be one of the questions we will have to address in the near future. In addition, there are still some limitations in our research. How CLDN10 is expressed on the outer membrane of mitochondrial, and how CLDN10 inhibits the binding of SIRT3 and ATP5O, these questions also need to be answered in the near future.

## Conclusions

All in all, the preset study has clarified the role of CLDN10 as a tumor suppressor gene in ccRCC, and for the first time illustrates the mechanism by which CLDN10 suppresses the growth and metastasis of ccRCC, which will provide important theoretical support for the exploitation of a novel therapeutic target to inhibit the progression of ccRCC.

## Supplementary Material

Supplementary figure.

## Figures and Tables

**Table 1:** The clinicopathologic characteristics of 122 mccRCC patients

Clinicopathologic characteristics	n (%)
**Age**	
≤65	77 (63.1)
>65	45 (36.9)
**Gender**	
Male	91 (74.6)
Female	31 (25.4)
**Tumor size**	
<5 cm	23 (18.8)
≥2 cm, <10 cm	60 (49.2)
≥10 cm	39 (32.0)
**Tumor stage**	
T1	21 (17.2)
T2	16 (13.1)
T3	81 (66.4)
T4	4 (3.3)
**Histological grade**	
G1	3 (2.5)
G2	36 (29.5)
G3	64 (52.4)
G4	19 (15.6)
**Overall survival**	
Alive	41 (33.6)
Dead	81 (66.4)
**Replase free survival**	
Non-replased	40 (72.2)
Replased	82 (27.8)

**Table 2:** Univariate and multivariate Cox regression analysis of OS in 122 mccRCC patients

Parameters	Univariate analysis				Multivariate analysis			
	P	HR	95%CI		P	HR	95%CI	
			Lower	Upper			Lower	Upper
**Age**								
≤65 (n=77)		1.0000						
>65 (n=45)	0.1580	1.3780	0.8833	2.1490				
**Gender**								
Female (n=31)		1.0000						
Male (n=91)	0.6140	1.1450	0.6772	1.9350				
**Tumor size**								
<10 cm (n=83)		1.0000				1.0000		
≥10 cm (n=39)	**0.0431**	1.6190	1.0150	2.5840	**0.0436**	1.6660	1.0148	2.7350
**Tumor stage**								
T1/T2 (n=37)		1.0000				1.0000		
T3/T4 (n=85)	**0.0325**	1.7190	1.0460	2.8250	0.7762	1.0840	0.6234	1.8830
**Histological grade**								
Grade 1-3 (n=103)		1.0000						
Grade 4 (n=19)	0.0789	1.7520	0.9373	3.2760				
**CLDN10**								
high expression (n=61)		1.0000				1.0000		
low expression (n=61)	**<0.0001**	4.0750	2.5090	6.6190	**<0.0001**	4.0860	2.4737	6.7490

**Table 3:** Univariate and multivariate Cox regression analysis of DFS in 82 mccRCC patients

Parameters	Univariate analysis				Multivariate analysis			
	P	HR	95%CI		P	HR	95%CI	
			Lower	Upper			Lower	Upper
**Age**								
≤65 (n=54)		1.0000				1.0000		
>65 (n=28)	**0.0046**	2.2110	1.2780	3.8240	**0.0156**	2.0570	1.1460	3.6900
**Gender**								
Female (n=26)		1.0000						
Male (n=56)	0.4120	1.2920	0.7005	2.3830				
**Tumor size**								
<10 cm (n=50)		1.0000						
≥10 cm (n=32)	0.1190	1.5440	0.8937	2.6660				
**Tumor stage**								
T1/T2 (n=18)		1.0000				1.0000		
T3/T4 (n=64)	**0.0011**	3.6820	1.6830	8.0560	0.1337	1.9550	0.8140	4.6960
**Histological grade**								
Grade 1-3 (n=68)		1.0000						
Grade 4 (n=14)	0.2910	1.4780	0.7157	3.0520				
**CLDN10**								
high expression (n=41)		1.0000				1.0000		
low expression (n=41)	**<0.0001**	4.8360	2.6410	8.8580	**<0.0001**	4.3680	2.2800	8.3700

## Abbreviations

ccRCC: clear cell renal cell carcinoma
TJs: tight junctions
CLDN10: Claudin-10
TCGA-KIRC: The Cancer Genome Atlas-Kidney Renal Clear Cell Carcinoma
OS: Overall Survival
DFS: Disease Free Survival
Co-IP: Co-immunoprecipitation
TMT: Tandem mass tags
ATP5O: ATP synthase subunit O, mitochondrial
SIRT3: NAD-dependent protein deacetylase sirtuin-3, mitochondrial
NDUFS2: NADH dehydrogenase [ubiquinone] iron-sulfur protein 2, mitochondrion inner membrane
ROS: reactive oxygen species
NAD+: Nicotinamide adenine dinucleotide
