# Supplementary material for: Claudin-10 overexpression suppresses human clear cell renal cell carcinoma growth and metastasis by regulating ATP5O and causing mitochondrial dysfunction
Source: Int J Biol Sci. 2022 Mar 6;18(6):2329–44. doi: 10.7150/ijbs.70105 (PMC8990465; doi:10.7150/ijbs.70105)

Supplementary figure 1: The correlation of CLDN10 expression with the expression of five key OXPHOS complexes. **A**, The correlation between CLDN10 expression and the expression of NDUFB8, SDHB, UQCRC2, MT-CO2 and ATP5A1. **B**, Prognostic value of SDHB in ccRCC patients. These data are all from TCGA-KIRC.

Supplementary figure 1

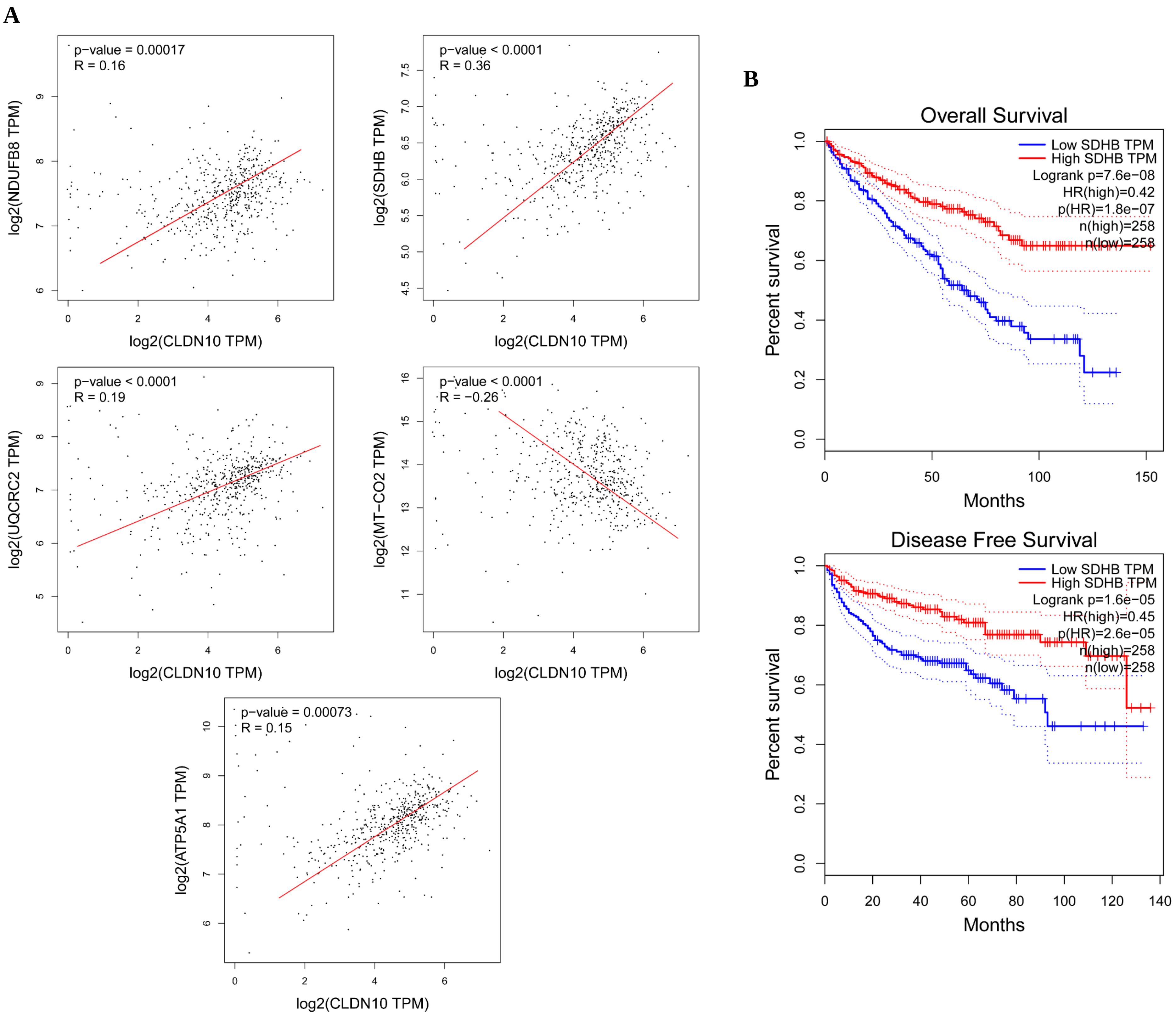

Supplement: Supplementary file 1 — Supplementary figure. [file ijbsv18p2329s1.pdf]
